# Azole Drugs Are Imported By Facilitated Diffusion in *Candida albicans* and Other Pathogenic Fungi

**DOI:** 10.1371/journal.ppat.1001126

**Published:** 2010-09-30

**Authors:** Bryce E. Mansfield, Hanna N. Oltean, Brian G. Oliver, Samantha J. Hoot, Sarah E. Leyde, Lizbeth Hedstrom, Theodore C. White

**Affiliations:** 1 Seattle Biomedical Research Institute, Seattle, Washington, United States of America; 2 Brandeis University Department of Biology and Chemistry, Waltham, Massachusetts, United States of America; 3 University of Washington Program in Pathobiology, Seattle, Washington, United States of America; University of California San Francisco, United States of America

## Abstract

Despite the wealth of knowledge regarding the mechanisms of action and the mechanisms of resistance to azole antifungals, very little is known about how the azoles are imported into pathogenic fungal cells. Here the *in-vitro* accumulation and import of Fluconazole (FLC) was examined in the pathogenic fungus, *Candida albicans*. In energized cells, FLC accumulation correlates inversely with expression of ATP-dependent efflux pumps. In de-energized cells, all strains accumulate FLC, suggesting that FLC import is not ATP-dependent. The kinetics of import in de-energized cells displays saturation kinetics with a *K_m_* of 0.64 uM and *V_max_* of 0.0056 pmol/min/10^8^ cells, demonstrating that FLC import proceeds via facilitated diffusion through a transporter rather than passive diffusion. Other azoles inhibit FLC import on a mole/mole basis, suggesting that all azoles utilize the same facilitated diffusion mechanism. An analysis of related compounds indicates that competition for azole import depends on an aromatic ring and an imidazole or triazole ring together in one molecule. Import of FLC by facilitated diffusion is observed in other fungi, including *Cryptococcus neoformans*, *Saccharomyces cerevisiae*, and *Candida krusei*, indicating that the mechanism of transport is conserved among fungal species. FLC import was shown to vary among *Candida albicans* resistant clinical isolates, suggesting that altered facilitated diffusion may be a previously uncharacterized mechanism of resistance to azole drugs.

## Author Summary

Azole antifungals are used to treat a wide variety of fungal infections of humans, animals and plants. A great deal is known about how the azoles interact with their target enzyme within fungal cells and how the azoles are exported from the fungal cell through various efflux pumps. Altered interactions with the target enzyme and altered efflux pump expression are common mechanisms of azole resistance in fungi. However, the mechanism by which azoles enter a fungal cell is not clear—many have assumed that azoles passively diffuse into the cell. This study demonstrates that azoles are not passively diffused, or actively pumped, into the cell. Instead, azoles are imported by facilitated diffusion, mediated by a transporter. Facilitated diffusion of azoles is saturable. All clinically important azoles, and many structurally related compounds, compete for FLC import, while structurally unrelated drugs do not compete. Azole import by facilitated diffusion is shown in four species of fungi, suggesting that it is common for most if not all fungi. Altered facilitated diffusion is observed in a collection of clinical isolates, suggesting that altered import is a previously uncharacterized mechanism of resistance.

## Introduction

The incidence of invasive fungal disease has increased over 200% in the US in the last 25 years [1], likely the result of a parallel increase in the immunocompromised patient population. *Candida* species are the most common invasive fungal pathogens, with *Candida albicans* accounting for more than 50% of all infections [2]. *C. albicans* causes oral, vaginal and systemic disease, with the highest morbidity rate (30%–50%) occurring with systemic *Candida* infections in neutropenic transplant patients [3]–[5].

One of the first lines of defense for treating pathogenic fungal infections are the azole drugs, including FLC, the most commonly used azole. FLC and other azoles affect the biosynthesis of ergosterol (the major sterol in the fungal plasma membrane) by inhibiting 14α lanosterol demethylase, the product of the *ERG11* gene. The significant increase in invasive fungal infections and the prolonged and repeated treatment of AIDS patients has resulted in a marked increase in the emergence of FLC-resistant *C. albicans* isolates [6]–[8]. In *C. albicans*, several mechanisms of resistance have been well characterized (reviewed in [6]–[8]). However, clinical isolates have not been investigated for altered azole import as a mechanism of resistance.

Several studies have investigated the accumulation of drugs in resistant clinical isolates of *C. albicans* [7], [9]–[12]. These studies show reduced intracellular FLC in the isolates, which is energy dependent and the result of overexpression of the major facilitator pump gene *MDR1*, and the ABC transporter efflux pump genes *CDR1* and *CDR2* [7], [8]. Both Cdr1p and Cdr2p are ATP-dependent efflux pumps, whereas Mdr1p utilizes proton motive force at the membrane to transport drugs and other compounds.

Surprisingly, the mechanism(s) by which FLC enters the *C. albicans* cell remain unstudied. Defects in drug import are a common mechanism of drug resistance in other pathogenic organisms, but to date, there have been no reports that *C. albicans* utilizes altered import as a resistance mechanism. Azoles are widely assumed to enter the fungal cell via passive diffusion [13]–[15], but there is little evidence to support this. Some evidence for facilitated diffusion of azoles has been reported, but these experiments were performed in energized cells in which drug efflux was active, and therefore failed to uncouple import and export [9], [16].

This study biochemically characterized the mechanism by which FLC is taken into *C. albicans* cells. The results suggest that the FLC enters the cell by energy-independent facilitated diffusion in *C. albicans* and other pathogenic fungi. In addition, import levels vary among resistant clinical isolates, suggesting that import is a previously uncharacterized mechanism of resistance to azole drugs in *C. albicans*.

## Results

### Fluconazole Uptake by *C. albicans*

**Figure 1:**
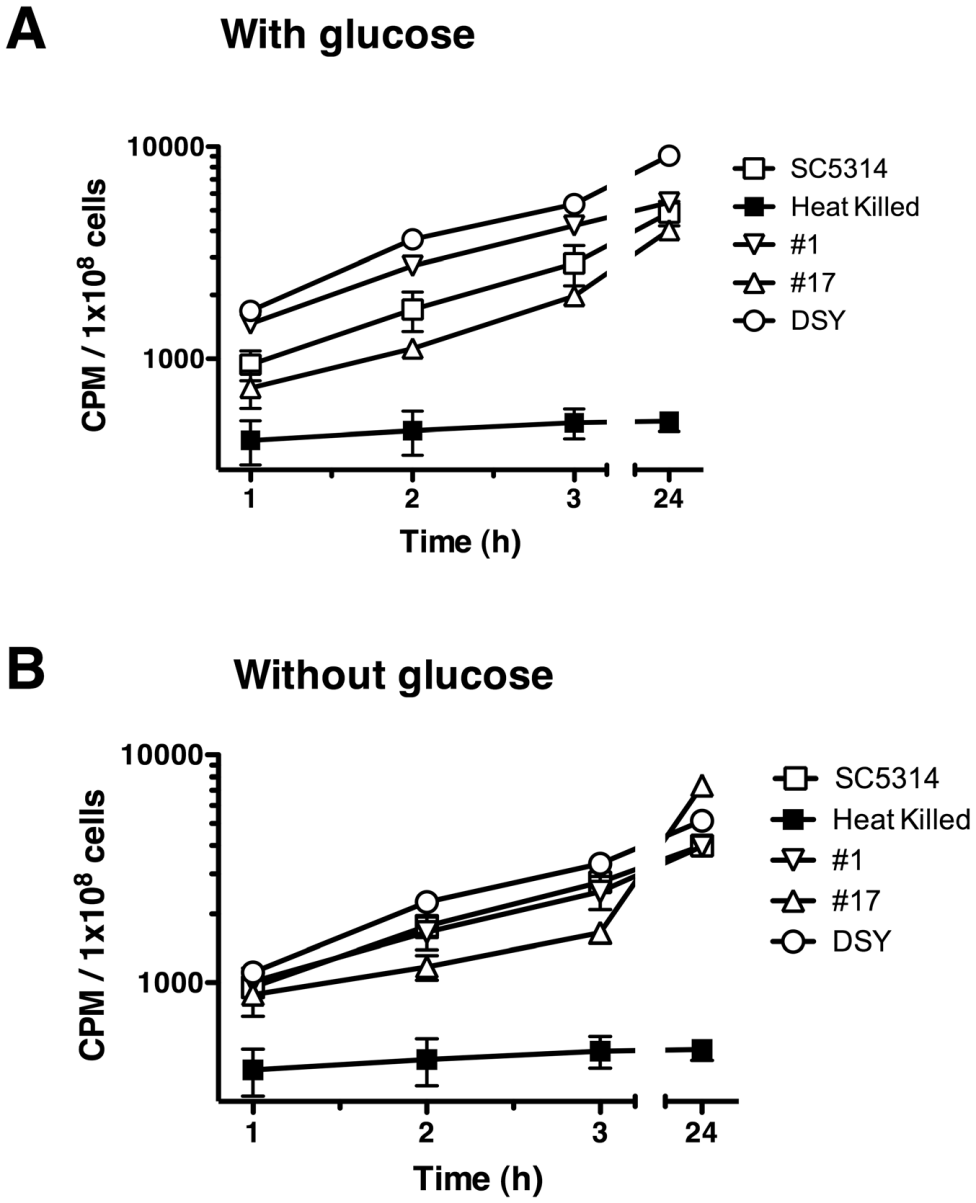
Effect of glucose on [^3^H]-FLC accumulation levels in *C. albicans*. A. FLC accumulation in the presence of 2% glucose. B. FLC accumulation in the absence of glucose after cells were starved for 2 h. Strains  =  SC5314 (open squares), #1 (inverted triangles), #17 (triangles) DSY1050 (open circles), heat-killed SC5314 (filled squares). Samples were removed at 1, 2, 3, and 24 h. Samples were normalized to CPM/1×10^8^ cells (y axis) and compared to the incubation time (x axis). Error bars are included and most times are smaller than the symbol.

The accumulation of [^3^H]-FLC was analyzed in energized cells in the presence of glucose (Fig. 1A), in which energy-driven importers and efflux pumps would have an effect on drug accumulation. Four *C. albicans* strains were tested: a wild type strain (SC5314), a susceptible clinical isolate (#1), a matched resistant clinical isolate that overexpresses *CDR1*, *CDR2* and *MDR1* (#17, ref [17], [18]) and a genetic construct strain DSY1050 that is deleted for *CDR1*, *CDR2* and *MDR1* [19]. Cells were incubated with [^3^H]-FLC and uptake was quenched by rapid dilution and filtration as described in Experimental Procedures. FLC accumulation was observed to be linear over 3 h, with maximum accumulation observed after 24 h (Fig. 1), as accumulation did not increase after 24 h (data not shown). In the presence of glucose, the expression of efflux pumps does have a minor effect on [^3^H]-FLC accumulation: strain #17, which overexpresses *CDR1*, *CDR2* and *MDR1*, shows the lowest accumulation, and strain DSY1040, which is deleted for the three pumps, shows the highest level of accumulation.

Since FLC accumulation in the presence of glucose will be the sum of both FLC import and export with ATP-dependent efflux pumps, the strains were tested after glucose starvation for 2 h (Fig. 1B). If import is energy-independent, the depletion of ATP should inactivate ATP-dependent efflux pumps and accumulation should largely be due to import. Under these conditions, there is less variation between strains in the accumulation of [^3^H]-FLC and accumulation still occurred at a similar rate (Fig. 1B). The addition of the glycolysis inhibitor 2-deoxy-D-glucose during the 2 h preincubation starvation did not alter [^3^H]-FLC accumulation levels, suggesting that the cells are indeed de-energized in the absence of glucose (data not shown). This suggests that accumulation does occur in de-energized cells and that efflux pumps do not have an effect on FLC accumulation in the absence of glucose.

Cells were inactivated to determine if import depends on living cells. [^3^H]-FLC did not accumulate when SC5314 cells were killed by heat (70°C for 45 m; Fig. 1 and Table 1, row 1) or methanol treatment (95% methanol for 45 m, Table 1, row 1). The heat-killed and methanol-killed cells appeared intact when observed under light microscopy. However, live cells were impermeable to propidium iodide (<1.0% of cells were stained) while heat-killed or methanol-killed cells were permeable to propidium iodide (98.8% and 99.5% cells stained respectively), suggesting that the lack of accumulation in killed cells is due to permeable cell walls and/or membranes.

**Table 1:** Effect of Condition Changes on [^3^H]-FLC Import in SC5314.

Row	Conditions	Condition details	Import^a^	Relative change^b^
1	Killed Cells	Methanol	289±57	0.12
		Heat	470±45	0.20
2	Temperature	4°C	556±54	0.24
		25°C (WT)	2335±193	1.00
		30°C	2845±130	1.22
		37°C	2583±417	1.11
3	Growth	OD_600_ 0.4 (log)	23,394±1795	4.67
		OD_600_ 8–10 (post diauxic shift)(WT)	5007±656	1.00
4	Morphology	Germ tubes (Hyphae)	8826±462	3.78
		Yeast (WT)	2336±193	1.00
5	pH	pH 3	2754±96	1.00
		pH 5 (WT)	2766±164	1.00
		pH 7	2588±187	0.94
		pH 9	2299±339	0.83
6	Low Oxygen	Growth in micro-aerophilic conditions	11946±466	4.55
		Growth under normal conditions	2624±174	1.00

a Import was analyzed as described in the Materials and Methods. Values are CPM/1×10^8^ cells ± SE. Values are representative of three independent experiments. Conditions were analyzed independent of other conditions.

b Values are relative to import of live SC5314 (WT) under standard conditions at 24 h.

### Effect of Condition Changes on [^3^H]-FLC Import

Fig. 1 showed that all strains accumulate FLC in the absence of glucose. A variety of conditions that might have an affect on azole import were assayed for altered uptake of [^3^H]-FLC (Table 1). [^3^H]-FLC accumulation is temperature dependent, with minimal accumulation at 4°C and maximum accumulation at 30°C, with a slight reduction at 37°C (Table 1, row 2). This is inconsistent with passive diffusion of FLC, which should increase with an increase in temperature. Import per cell was also studied during various growth phases (Table 1, row 3) with maximum import occurring in cells growing in mid-log phase (OD_600_ 0.4). Germ tubes (hyphae) exhibited a 4 fold increase in [^3^H]-FLC accumulation when compared to yeast cells (Table 1, row 4), although cell numbers are difficult to determine for hyphal cells. Accumulation appears to be unaffected by changes in pH (Table 1, row 5). Surprisingly, the highest change in accumulation occurs under micro-aerophilic conditions, where cells accumulate over 4 fold more than cells growth with normal aeration (Table 1, row 6). The changes in accumulation associated with growth and oxygen levels, and the reduced import at higher temperatures confirms that accumulation is not simply passive diffusion.

### Kinetics of [^3^H]-FLC Import

**Figure 2:**
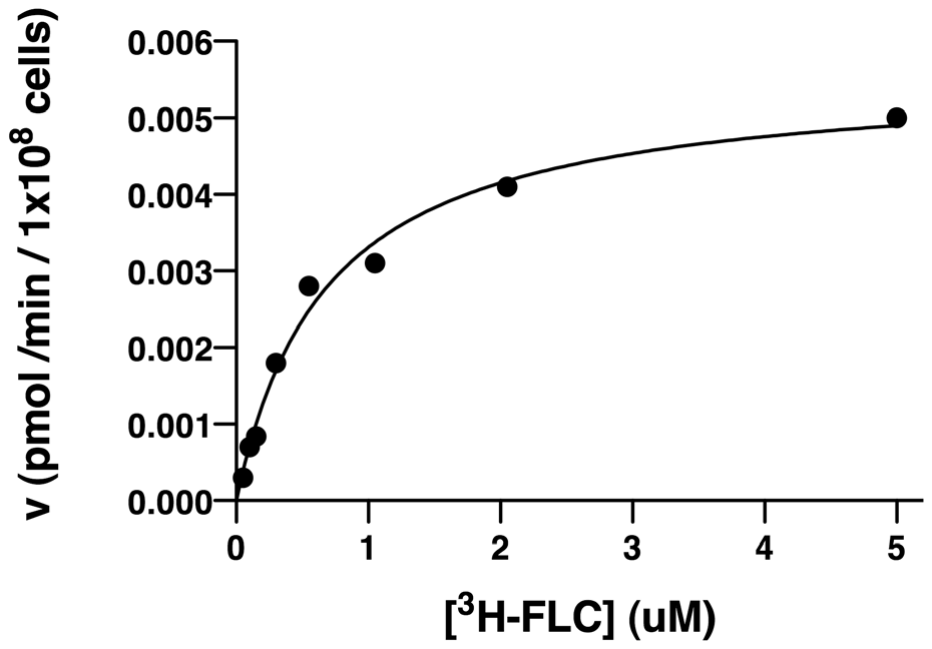
Kinetics of import of [^3^H]-FLC. Import kinetics measure the initial accumulation rate (y axis) as a function of [^3^H]-FLC concentration (x axis), using *C. albicans* strain SC5314. The Michaelis-Menten equation was used to determine *K_m_* of 0.64 uM and *V_max_* of 0.0056 pmol/min/1×10^8^ cells. Results are a representative graph of a minimum of three independent experiments.

The high level accumulation of [^3^H]-FLC in de-energized cells (Fig. 1B) and the changes in accumulation with changes in the environment (Table 1) suggest FLC import is carried out by facilitated diffusion. Therefore, the kinetics of FLC import were assayed in detail for de-energized SC5314 cells, studying the initial rate of import across a spectrum of FLC concentrations (Fig. 2). FLC import displayed saturation kinetics with a *K_m_* of 0.64 uM and *V_max_* of 0.0056 pmol/min/10^8^ cells. The saturation kinetics strongly support facilitated diffusion through a specific transporter as the mechanism of FLC import.

### Competition of [^3^H]-FLC Import by Other Azoles

**Figure 3:**
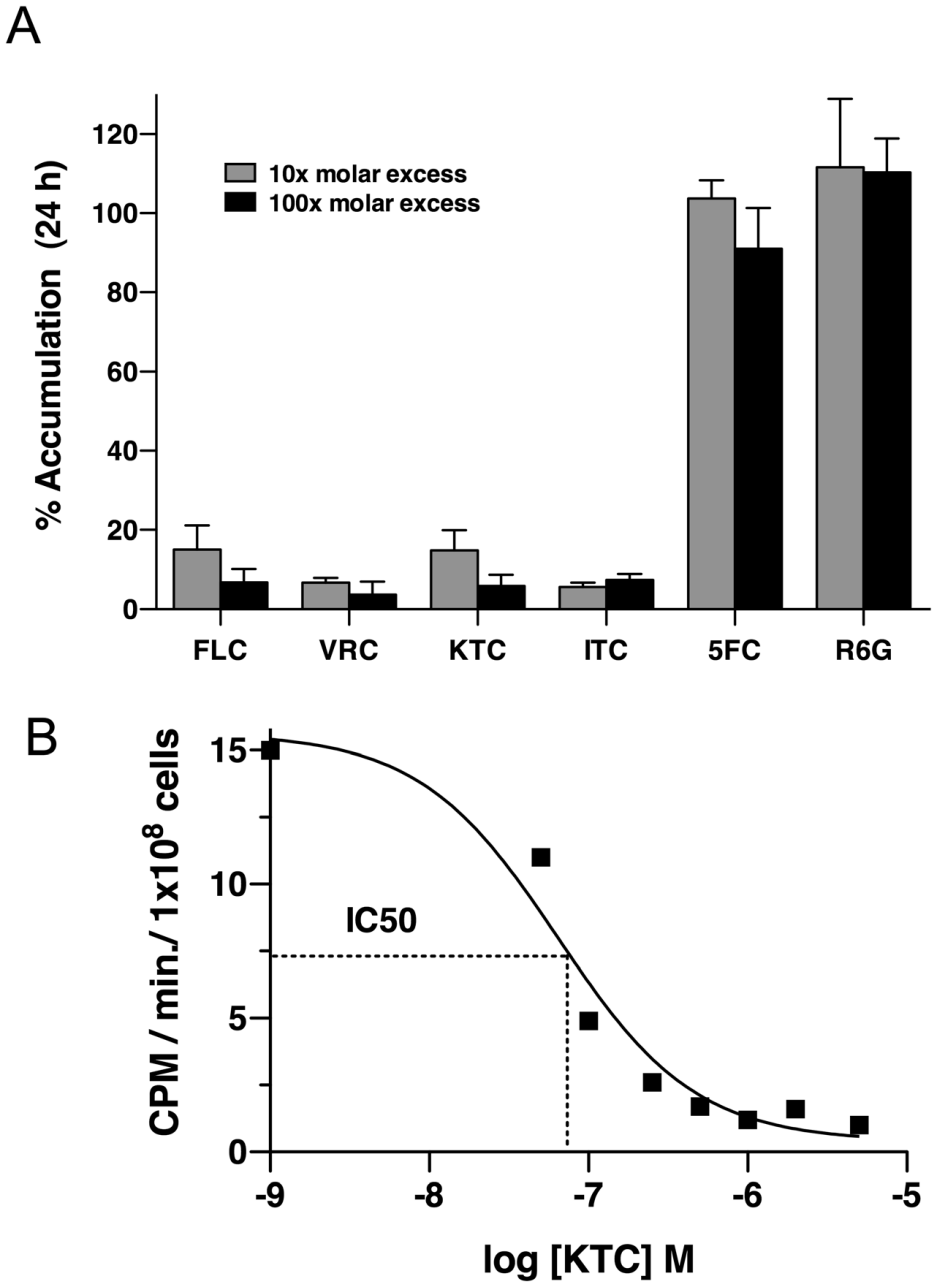
Imidazoles and triazoles compete for [^3^H]-FLC uptake, but 5FC and R6G do not. Samples were grown and processed as outlined in Experimental Procedures. (A) Influx was tested in the presence of 10 x (grey bars) and 100 x (black bars) molar excess of unlabeled azoles, 5FC or R6G. Import of [^3^H]-FLC was measured at 24 h post incubation. Values are the average of three biological replicates with standard deviations, shown as a percentage of SC5314 import in the absence of unlabeled compounds. All azoles compete at a significant level at both 10X and 100X (*p*<0.05). (B) To quantify competition for [^3^H]-FLC import, increasing concentrations of unlabeled compound (KTC) were added to the incubation mixture and samples were analyzed for [^3^H]-FLC import at 10 m, 30 m, 60 m and 180 m. Using linear regression analyses, each square represents the rate of [^3^H]-FLC uptake in the presence of the unlabeled KTC. The kinetics are a measure of rate of import of [^3^H]-FLC (y axis) as a function of the log of unlabeled KTC concentration (x-axis). Dotted line graphically represents the calculation of IC_50._

To determine whether other azole drugs utilize the same transporter as FLC, import of [^3^H]-FLC was assayed in de-energized SC5314 cells in the presence of unlabeled azole drugs including FLC, ketoconazole (KTC), voriconazole (VRC), and itraconazole (ITC). As seen in Fig. 3A, all four azoles inhibited the import of [^3^H]-FLC at 10 fold and 100 fold excess, suggesting that the same transporter is involved in the uptake of all four azole drugs.

50% inhibitory concentration (IC_50_) values were calculated for KTC and ITC by measuring the rate of [^3^H]-FLC import in the presence of excess unlabeled KTC or ITC. The calculated IC_50_ values are 65 nM and 48 nM for KTC and ITC respectively (Fig. 3B and data not shown). As 50 nM of [^3^H]-FLC is used in the assay, the IC_50_ values suggest that FLC, KTC and ITC compete at equimolar concentrations for import into the cell, suggesting that they compete equivalently as substrates for the transporter(s).

The antifungal 5-flucytosine (5FC) and the fluorescent dye rhodamine 6G (R6G) have been hypothesized to share the same import mechanism as FLC [10], [20]. 5FC is a nucleoside analog [20] and R6G is a dye known to be effluxed by Cdr1p and Cdr2p [10]. However, neither 5FC (5 µM) nor R6G (5 µM) at 100 fold molar excess reduced the import of [^3^H]-FLC (Fig. 3A). These observations suggest that 5FC and R6G have independent import mechanisms, and that the assay does not measure drug export, since R6G acts as a substrate for the efflux pumps.

### Competition with Other Compounds

As FLC is unlikely to be the natural substrate for the import mechanism, additional compounds were tested as competitors for FLC import. The following types of compounds were tested in molar excess and were shown not to compete with FLC: sugars, nucleosides, amino acids, unrelated antifungals, salts and unrelated drugs (complete list of compounds is in Table 2).

**Table 2:** Compounds Unrelated to FLC That Do Not Compete for Inhibition of FLC Import.

Sugars	Inositol, Mannose, Galactose, Glucose, Sucrose, beta 1–3 glucan (laminarin)
Nucleosides/Bases	Adenosine, Thymidine, Guanosine, Cytosine, Uracil
Amino acids	Cas-amino acids, Arg, Pro, His (H), Phe (F)
Oligopeptides	HF, FH, HAF, FAH, HAAF, FAAH, HAAAF, FAAAH
Non-azole antifungals	5FC, Amphotericin B, Terbinafine, Fenpropimorph
Polyamines	Spermidine, Spermine, Ornithine
Sterol Related	Ergosterol, Farnesol, Dialkyl imidazole STN54^a^
Salts	CaCl_2_, CuSO_4_, FeSO_4_, K_2_SO_4_, MgSO_4,_ NaCl, and NH_4_SO_4_
Unrelated Drugs	DNP (2,4-Dinitrophenol, oxidative phosphorylation inhibitor), CCCP (Carbonyl cyanide m-chlorophenyl hydrazone, oxidative phosphorylation inhibitor), Brefeldin A (transport inhibitor), Rhodamine 6G (competes for efflux pumps)

All compounds were tested in the standard assay at 100x molar excess to [^3^H] FLC and had no effect.

a STN54 is a dialkylimidazole with very potent activity against C14-demethylease, the target of the azole drugs [24].

It is known that uptake of ergosterol increases under low-oxygen conditions in *Saccharomyces cerevisiae* [21]. FLC import also increases under low-oxygen conditions (Table 1, row 6). These observations suggested that FLC and ergosterol might share an import mechanism. However, 10 and 100 fold excess of unlabeled ergosterol did not compete for [^3^H]-FLC import in this assay (data not shown).

In analyzing the competition data with FLC, ITC, KTC and VRC (Fig. 3A), it became evident that all of the azoles share two structural moieties in common – a) a halogenated aromatic 6 carbon ring and b) an imidazole [2 nitrogen (N)] or triazole (3 N) 5-member ring (Table 3, structures shown in Supplemental Figure S1 based on the compound structures in the NIH PubChem Compound Database [22]). Using this as a starting point, we have expanded our understanding of the structural components that are required to compete for FLC import (Table 3). Rows 1 to 8 show the molecules that compete for FLC import, including the clinically important FLC, VRC, ITC, KTC, and posaconazole, as well as a fluorescein labeled ITC, and two agricultural azoles, paclobutrazol and azaconazole. All of these compounds contain a 6-carbon ring halogenated with fluorine (F) or chlorine (Cl) and an imidazole (2N) or triazole (3N) 5-member ring. Importantly, fluorescein labeled ITC competes for FLC import, but it has no antifungal activity on its own (data not shown). Many of these compounds contain additional ring structures within the molecule (Table 3 and Supplemental Figure S1).

**Table 3:** Compounds Related to FLC and Assayed for Inhibition of FLC Import.

Row	Compound	Aromatic 6 carbon ring	5 member ring	Additional rings	Competition^a^
1	FLC	Halogenated (2 F)	Triazole (2x)	-	+
2	VRC	Halogenated (2 F)	Triazole	+	+
3	ITC	Halogenated (2 Cl)	Triazole	+	+
4	Fluorescein-labeled ITC^b^	Halogenated (2 Cl)	Triazole	+	+
5	Posaconazole	Halogenated (2 F)	Triazole	+	+
6	Paclobutrazol	Halogenated (1 Cl)	Triazole	-	+
7	KTC	Halogenated (2 Cl)	Imidazole	+	+
8	Azaconazole	Halogenated (2 Cl)	Imidazole	+	+
9	Tipifranib^c^	Halogenated (2 ring, 1 Cl each)	Methylated Imidazole	+	-
10	Tipifarnib 2g^c^	Halogenated (2 ring - 1 Cl, and 1 Cl/methylated)	Methylated Imidazole	+	-
11	STN54^d^	No halogenation (2 ring)	Modified Imidazole	+	-
12	Benznidazole	No halogenation	Modified Imidazole	-	-
13	Difluoro-benzene	Halogenated (2 F)	No ring	-	-
14	Imidazole	No ring	Imidazole	-	-
15	Difluoro-benzene and Imidazole	Halogenated (2 F)	Imidazole	-	
16	His	No ring	Imidazole	-	-
17	Phe	No halogenation	No ring	-	-

a All compounds were tested at 100x molar excess to FLC for their effect

b Fluorescein-labeled itraconazole was prepared by J. Liu and collaborators (unpublished data), and has no anti-*Candida* activity (T. White and collaborators, unpublished data).

c Tipifarnib, an anti-cancer drug and the Tipifarnib analog JK11 (compound 2g) are both potent inhibitors of C14-demethylase, the target of the azole drugs [23].

d STN54 is a dialkyl-imidazole with very potent activity against C14-demethylease, the target of the azole drugs [24].

Four other drugs were tested because they are active against the sterol biosynthetic pathway and have antimicrobial activities, including Tipifarnib, the Tipifarnib derivative 2g [23], STN54 [24], and Benznidazole. None of these molecules compete (Table 3, rows 9–12). All four have a modified imidazole rings, and two have aromatic rings without halogenation, suggesting that one or both of these two structures are important for competition of import.

To analyze these two components separately, the compounds difluoro-benzene and imidazole were tested in the import assay independently and together (Table 3 rows 13–15). The compounds individually or in combination did not compete, suggesting that a compound must have both moieties physically linked to compete for FLC import.

Finally, the imidazole ring resembles histidine (H) and the aromatic ring resembles phenylalanine (F). These two amino acids alone do not compete for FLC import (Table 3, rows 16–17). To test if physical linkage of H and F would compete for FLC import, oligopeptides were prepared including the dipeptides HF and FH, and larger peptides separated by one, two or three alanines (A) (Table 2). None of these oligopeptides competed for FLC import. To address concerns about peptide degradation, the peptides were tested for competition of FLC import in the presence of protease inhibitors, and at vast molar excess (10,000X) with no appreciable competition.

### Import of [^3^H]-FLC in Other Pathogenic Fungi

FLC and other azoles are used in the treatment of many pathogenic fungi. De-energized *Cryptococcus neoformans*, *S. cerevisiae* and *C. krusei*, which is an intrinsically azole resistant *Candida* species, were all tested for import of [^3^H]-FLC and import levels were compared to [^3^H]-FLC import of *C. albicans* (SC5314). As shown in Table 4, each species was able to import and accumulate [^3^H]-FLC at levels similar to *C. albicans*. Similar experiments with equivalent OD units of *E. coli* cells did not detect FLC import above background (data not shown), supporting the conclusion that FLC import is not by passive diffusion. Collectively, these data suggest that azoles are imported by a specific transporter that may be conserved across fungal species.

**Table 4:** Import of [^3^H]-FLC in Other Pathogenic Fungi Compared to *C. albicans*.

Species	Import^a^	Relative change^b^
*Cryptococcus neoformans*	5604±277	3.22*
*Candida krusei*	3802±203	2.19*
*Saccharomyces cerevisiae*	2283±322	1.31
*Candida albicans*	1737±66	1.00

a Import was analyzed as described in the Materials and Methods. Values are CPM/1×10^8^ cells ± SE. Values are representative of three independent experiments.

b Values are relative to import of *Candida albicans* under standard conditions at 24 h. * = *p*<0.05 relative to *C. albicans*.

### *Saccharomyces* Gene Deletion Screen

*S. cerevisiae* shows FLC import similar to *C. albicans* (Table 4). To ensure that known pumps are not involved in import, two *S. cerevisiae* strains, AD1-8 and AD1-9 [25], [26], which are deleted for eight and nine efflux pumps respectively, were tested for import. Import was not significantly different from the wild type strain (data not shown).

**Figure 4:**
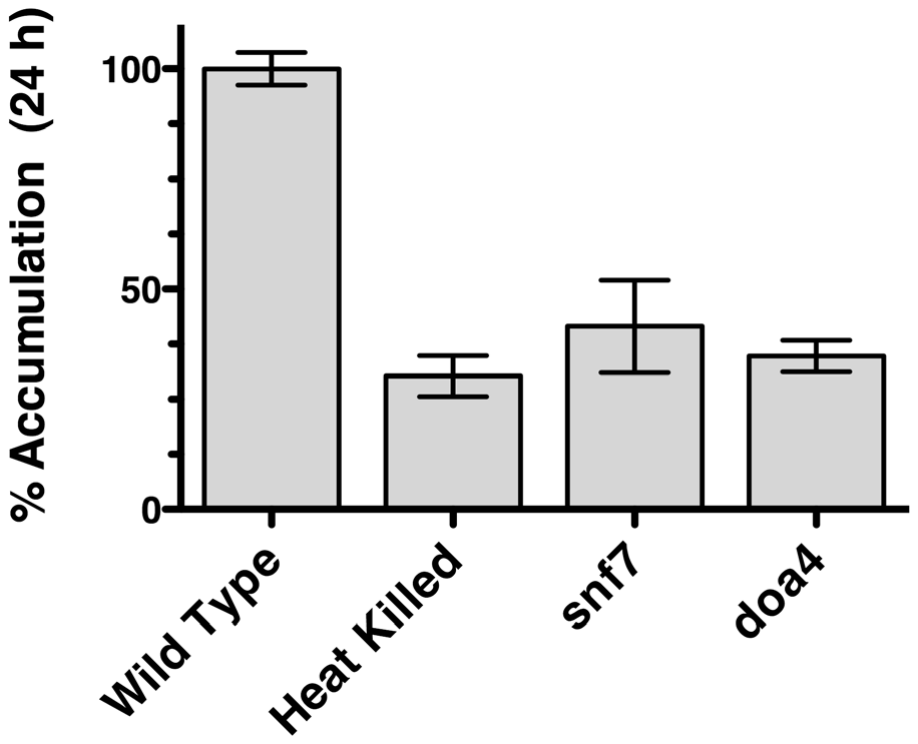
[^3^H]-FLC import in *S. cerevisiae* mutants. FLC import was measured at 24 h for strains S288C (wild type), gene deletion *snf7*, gene deletion *doa4*, and heat killed S288C. Import is expressed relative to import in the wild type strain. Data is average of three biological replicates. *snf7*, *doa4*, and heat killed cells are all significantly reduced compared to wild type (*p*<0.05).

A collection of over 5,000 *Saccharomyces* strains containing deletions in non-essential genes is available to the research community [27]. The collection was screened biochemically, using a 96 well format (see Materials and Methods). Two gene deletions from the collection were identified that had significantly reduced fluconazole import (Fig. 4). *SNF7* and *DOA4* are involved in protein transport at the plasma membrane (reviewed in [28], [29]). *SNF7* is a member of the ESCRT III complex (Endosomal Sorting Complex Required for Transport) that is involved in recycling or degrading membrane proteins. *DOA4* is a de-ubiquitination enzyme involved in the same process and physically interacts with *SNF7*. As these proteins are both cytoplasmic and are involved in surface protein processing, they are not likely to be directly involved in import, but might be involved in subsequent processing of the import protein. Other ESCRT proteins in the screen did not have a significant loss of FLC import (data not shown).

**Figure 5:**
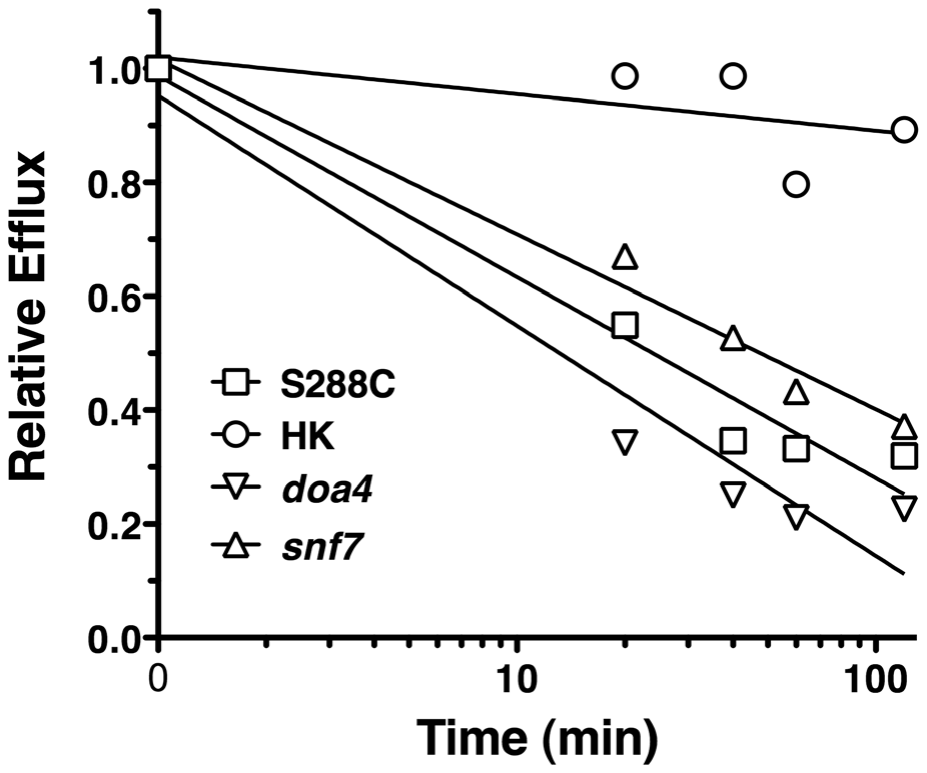
R6G Efflux of *S. cerevisiae* mutants. R6G efflux was measured over time for strains S288C (wild type, squares), gene deletion *snf7* (triangles), gene deletion *doa4* (inverted triangles), and heat killed S288C (circles). At each time point, 10,000 cells were measured for geometric mean fluorescence. Data is representative of three independent experiments. Data is expressed as fluorescence relative to the mean at t = 0. The relative mean fluorescence (y axis) is plotted against time in minutes (x axis).

One explanation for the role of *SNF7* and *DOA4* in FLC import is that they may interfere with degradation of efflux pumps, resulting in increased efflux. To test for efflux pump activity in *snf7* and *doa4* strains, R6G efflux was monitored over time (Fig. 5). Mutant strains of *snf7* and *doa4* effluxed R6G at similar rates to the wild type cells, while heat killed cells did not efflux R6G. The similar rates of efflux for wild type and *snf7* and *doa4* mutants indicate that reduced FLC import in these mutants (Fig. 4) is not the result of increased efflux, even through the assay was preformed with deenergized cells.

In *C. albicans*, the *SNF7* and *DOA4* mutants did not show altered FLC import when compared to wild type strains (data not shown). This suggests that the role of *SNF7* and *DOA4* is not conserved between the two species. It is not unusual for genes to have different functions between the two species (*i.e.* [30]–[33]). It also indicates that the role of *SNF7* and *DOA4* in *S. cerevisiae* is not central to the mechanism of FLC import.

### Import of [^3^H]-FLC in Azole Resistant Clinical Isolates

**Figure 6:**
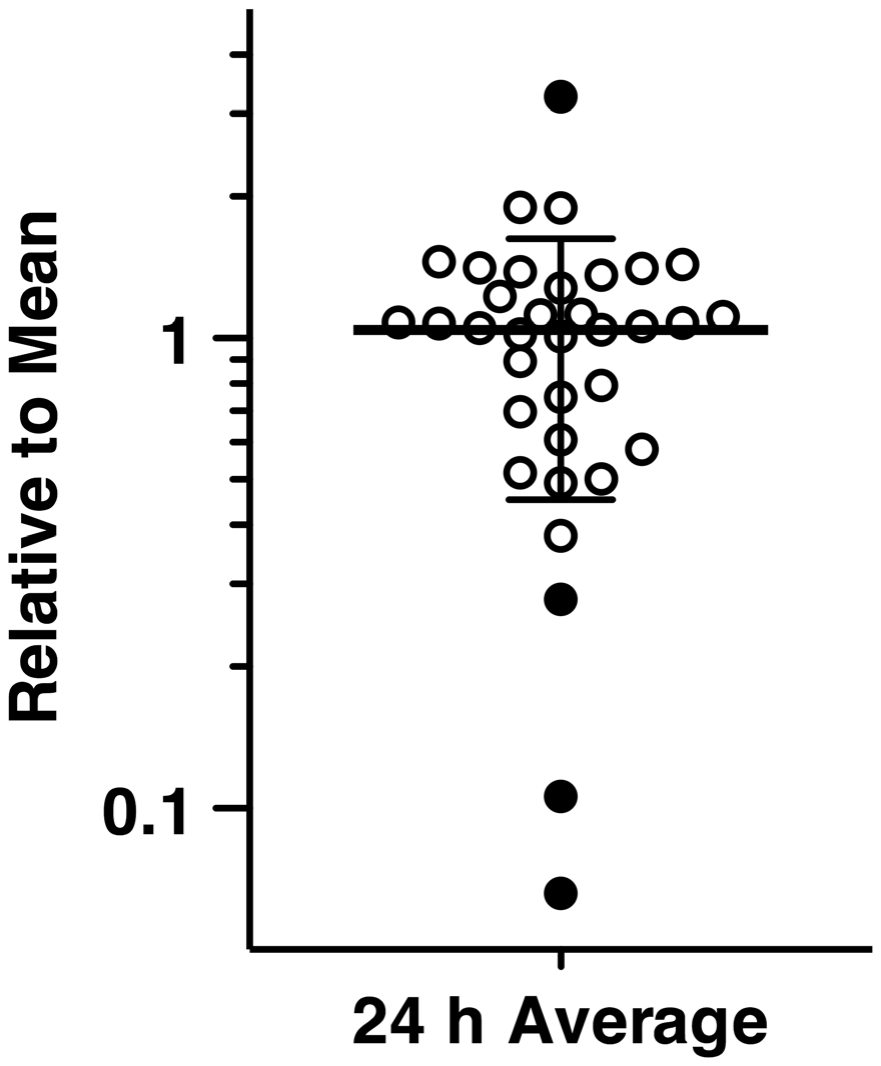
[^3^H]-FLC import of FLC resistant clinical isolates. Each circle represents the [^3^H]-FLC import of individual clinical isolates relative to the mean (y axis) at 24 h (x axis). Filled circles represent isolates that vary significantly from the mean (long horizontal line) plus standard deviations (shorter horizontal lines). Data is an average of four independent experiments.

To date, changes in azole import have not been reported as a mechanism of antifungal resistance in *C. albicans* or other pathogenic fungi. However, it is plausible that a mutation in the azole transporter would lead to azole resistance. A collection of unmatched clinical isolates of *C. albicans* was tested for their ability to import [^3^H]-FLC in the absence of glucose. Approximately 50% of the resistant isolates in this collection have unknown mechanisms of resistance [34]. As seen in Fig. 6, of the 35 isolates tested, four exhibited statistically significant alterations in [^3^H]-FLC import when compared to the median import value for all 35 isolates. Three of the isolates revealed significant decreases in import, while one had a significant increase. In addition, there is considerable variation in import between the other clinical isolates, both above and below the mean. Of the isolates exhibiting significantly decreased import, all exhibited other known but diverse mechanisms of resistance, including mutations in *ERG11* and overexpression of *CDR1*, *CDR2* (both of which are inactive in the import assay) or *MDR1*. The isolate with significantly increased import had no known mechanism of resistance. It is possible that these strains have altered import as well as other mechanisms of azole resistance, as it is not uncommon for clinical isolates to have multiple mechanisms of resistance [7], [34].

## Discussion

This study is the first to demonstrate and characterize facilitated diffusion of FLC into *C. albicans*. This is achieved using biochemical analyses. To date, FLC interactions with its target enzyme and FLC efflux from the cell have been studied in detail, but it was not known how FLC enters the fungal cell. With increasing resistance to azole therapies, it is increasingly important to understand mechanisms of FLC import and the contribution of that import to clinical resistance.

### Evidence of Facilitated Diffusion

The biochemical analyses clearly demonstrate that FLC import into *C. albicans* is not the result of passive diffusion but the result of facilitated diffusion. The biochemical evidence includes the following:

1. Inactivated cells do not accumulate FLC, no matter the method of inactivation: heat killing or methanol treatment (Fig. 1 and Table 1). Passive diffusion should accumulate drug whether the cell is living or dead.
2. The kinetics of FLC accumulation (Fig. 2) are not linear. Linear accumulation with increasing drug concentration would be expected for passive diffusion. However, the FLC accumulation displays saturation kinetics with a *K_m_* of 0.64 uM (0.2 ug/ml) and *V_max_* of 0.0056 pmol/min/1×10^8^ cells, strongly indicating that FLC import proceeds via facilitated diffusion. At low concentrations, the importer transports FLC into the cell at an increasing rate as the FLC concentration increases, but at higher FLC concentrations, the transporter becomes saturated and the rate of import levels off. The *K_m_* of 0.2 ug/ml is below the minimum inhibitory concentration (MIC) of the strain to FLC (1 ug/ml).
3. The final intracellular concentration of [^3^H]-FLC does not exceed the extracellular concentration. This is consistent with facilitated diffusion, and inconsistent with active transport, which can accumulate drug to levels above the extracellular concentration. Intracellular levels were estimated using the results from this study (Fig. 1) and estimated cell volumes of *Saccharomyces* cells [35]. The estimates suggest that the intracellular concentration for [^3^H]-FLC is 50 nM (data not shown), which is the extracellular concentration that is used in the assay.
4. Table 3 documents many compounds that will compete with FLC for import, as well as many compounds that do not compete. The ability for certain molecules to compete while others can not compete is strong evidence that there is a specific importer. Competition of specific compounds would not be expected with passive diffusion.

### FLC Import Is Not the Result of Drug Binding to the Cell Surface

There is strong biochemical evidence that FLC is not simply binding to the cells. First, the heat-killed and methanol-killed cells do not show FLC accumulation (Fig. 1 and Table 1). If the drug were binding to the cell wall, then the inactivated cells should also bind to drug. In fact, these treatments might expose more cell wall, resulting in more drug binding. However, FLC accumulation is not observed in these cells. Second, the cell wall component beta 1,3 glucan, available commercially as laminarin, does not compete for FLC binding (Table 2). It has been shown previously that cells in biofilms bind to FLC and this binding can be competed with laminarin [36]. However, laminarin had no effect on the FLC import assay used in this study (Table 2), confirming that the import assay and the biofilm assay are measuring separate phenomena.

### FLC Import Is Independent of Efflux Pumps

There is strong evidence that the results of these analyses are not the result of the efflux pumps. In the assay, FLC import kinetics were studied under de-energizing conditions (Fig. 2), in which the efflux pumps are not active, and the results were not altered by the addition of 2 deoxy-glucose (data not shown). In addition, the pump mutant DSY1050 in which *MDR1*, *CDR1* and *CDR2* are all deleted shows the same FLC import as wild type strains (Fig. 1). *Saccharomyces* strains deleted for 8 to 9 efflux pumps that are known to be associated with FLC efflux are still able to accumulate FLC (data not shown). Finally, inhibitors of mitochondrial function, including DNP and CCCP, that would eliminate function of *MDR1*, as well as *CDR1* and *CDR2*, had no effect on import (Table 2).

### General Azole Transporter

Azoles, including the clinically important FLC, KTC, ITC, VRC and POS, as well as the agriculturally important paclobutrazol and azaconazole, compete with labeled FLC for import (Fig. 3A, Table 3). The imidazole KTC and the triazole ITC compete at approximately equal molar concentrations (Fig. 3B and data not shown). This supports the conclusion that both imidazoles and triazoles utilize the same import mechanism as FLC. Unrelated antifungals, including terbinafine, fenpropimorph and amphotericin B, do not compete and thus are unlikely to use the same transporter.

### Structural Analysis

By testing related drugs, the structural moieties within a compound that allow it to compete with FLC for import were defined (Table 3). Compounds that compete contain both a halogenated (F or Cl) aromatic 6 member ring, and a triazole or imidazole 5 member ring. Compounds that do not compete have a methylation or other modification of the 5 member ring, potentially coupled to non-halogenated aromatic rings.

The 5 member triazole or imidazole ring is necessary for import, as compounds containing aromatic rings alone do not complete:. Similarly, neither His nor imidazole compete, consistent with the fact that neither contains an aromatic ring. The 6 member ring and the 5 member ring structures must be contained on the same molecule, as a mixture of difluoro-benzene and imidazole does not compete.

Given that His and Phe do not compete separately, it was of interest to test the two amino acids together. Oligopeptides containing His and Phe separated by 0 to 4 Ala did not compete for FLC import (Table 2) suggesting that linked His and Phe, which contain an imidazole ring and an aromatic ring, are not sufficient for recognition of the FLC import mechanism.

### 5FC and R6G

Both 5FC and R6G have been suggested to be co-transported with FLC. In a recent study, Noel et al. [20] characterized a series of *Candida lusitaniae* isolates that were cross-resistant to 5FC and FLC. The isolates tested were resistant to 5FC and susceptible to FLC unless the compounds were used simultaneously, in which case cross-resistance was observed. Noel *et al*. hypothesized that 5FC and FLC shared a common transporter and that extracellular 5FC was acting as a competitive inhibitor of FLC uptake transport [20]. Later reports indicated that the cross resistance was in fact due to mutations in genes encoding for cysteine permease [37] and cytosine deaminase [38] indicating that cross resistance is not due to a shared import mechanism. Data in this study support this conclusion as 5FC does not compete for [^3^H]-FLC import (Fig. 3).

R6G is a dye known to be effluxed from the cell by the pumps Cdr1p and Cdr2p. R6G has been shown to be capable of competing for FLC efflux [10]. It has therefore been hypothesized that FLC is also imported by Cdr1p or Cdr2p acting in reverse and that R6G could possibly compete for FLC import as well as export [10]. Data from this study clearly indicate that R6G does not compete for FLC import (Fig. 3).

### A Common Azole Transporter Conserved across Varying Fungal Species

Azoles are used to treat a wide variety of human (*Candida*, *Cryptococcus*, *Aspergillus*) and agricultural (*Pichia*, *Rhodoterula*, *Saccharomyces*) fungal pathogens [39]. Based on this widespread use, it was of interest to determine if other fungal species import and accumulate [^3^H]-FLC. It was shown that *C. neoformans*, *S. cerevisiae* and *C. krusei* import [^3^H]-FLC with similar kinetics (data not shown) and to final levels similar to *C. albicans* (Table 4). In addition the agricultural triazole paclobutrazol [40], [41] and the agricultural imidazole azaconazole [42], [43] compete with FLC for import (Table 3). These data suggest that the putative azole transporter is conserved across various fungal species. Interestingly, a recent study by Muller et al. [39] has shown that fungi found in an agricultural environment (including, but not limited to, various *Candida*, *Cryptococcus* and *Saccharomyces* species) are routinely treated with fluquinconazole, penconazole, tebuconazole or triadimenol. A significant portion of the isolates from different species that are resistant to these agricultural azoles were cross resistant to medical azoles including FLC, ITC, KTC or VRC. The cross-resistance could be the result of over-expression of the efflux pumps, but it is also possible that these isolates contain an alteration in an azole importer that is conserved across various fungal species and confers cross resistance to all azoles since all clinically significant azoles compete for FLC import (Table 3 and Fig. 3).

### Characterization of the *Saccharomyces* Gene Deletion Set for FLC Import

The biochemical screen for an importer in the haploid gene deletion strain collection failed to identify a potential import protein. The lack of a clear candidate suggests that the import protein is either an essential gene, which can not be deleted and would not be represented in the strain collection, or is present in more than one version - two paralogs with the same function or a gene family. In that case, deletion of one of the genes would not eliminate FLC import. It is possible that with two paralogs, import would be reduced 50% but that was not observed in the screen, despite the use of two different time points. If the gene family members or paralogs had substantially different kinetics, that would have been detected in the kinetic analysis. However, if the multiple copies of the importer all behave similarly, and the wild type strain has all of the genes expressed, then the differences would not be detected in the kinetic analysis. Further kinetic analysis awaits the identification of the import protein.

The biochemical screen did identify *SNF7* and *DOA4*, two components of the ESCRT III complex involved in recycling and degradation of surface proteins through the endosomes and multi-vesicular bodies (MVB). *SNF7* encodes one of the four subunits of the ESCRT III complexes, and *DOA4* encodes ubiquitin isopeptidase that is closely associated with the complex. However, the *C. albicans snf7* and *doa4* mutants did not exhibit a reduction in import, suggesting that the role of *SNF7* and *DOA4* in *S. cerevisiae* is not central to the import mechanism. The two gene deletion strains, *snf7* and *doa4*, do not have an altered MIC to FLC. However, the efflux pump *PDR5* is highly active in *Saccharomyces* wild type strains, and may mask any effect of *snf7* and *doa4* on FLC MIC. It is curious that other ESCRT proteins do not have an altered FLC import. Further work awaits the identification of the FLC import protein.

### Modified Import as a Potential Resistance Mechanism

To date, there has been no report of altered FLC import as a mechanism of antifungal resistance. The data in this study suggests that all azoles utilize the same import mechanism mediated by a transporter. Therefore, it is possible that a mutation in the putative transporter would lead to azole cross-resistance. 35 unmatched clinical isolates in which known mechanisms of resistance had been documented [34] were evaluated for FLC import (Fig. 6). Four of the 35 isolates exhibit significantly altered [^3^H]-FLC import. One of these four has no known mechanism(s) of resistance, while the other three are known to overexpress *ERG11*, *MDR1*, *CDR1* and/or *CDR2* or contain a mutation in *ERG11*. However, it is common for clinical isolates to exhibit multiple mechanisms of resistance [7], [8], [34]. Therefore, it is likely that these isolates have mutations that affect [^3^H]-FLC import, in addition to other mechanisms of resistance. These data suggest that loss, reduction, or alteration of azole import may be a previously unknown mechanism of antifungal resistance.

In conclusion, this study uses biochemical analyses to demonstrate that FLC import is not via passive diffusion but is in fact via facilitated diffusion. The data presented here represents the first comprehensive analysis of FLC import in *C. albicans*. This work also demonstrates that azoles share a common transport mechanism and azole import is conserved between several pathogenic fungi. Future directions will be focused on identifying and characterizing the protein responsible for this newly identified FLC facilitated diffusion.

## Materials and Methods

### Organisms and Growth Conditions

*Candida albicans* SC5314 (from W. Fonzi; [44]) is the wild type lab strain used in this study. *C. albicans* isolates #1 (2–76) and #17 (12–99) are a matched set of FLC susceptible and resistant clinical isolates in which #17 overexpresses *ERG11*, *CDR1*, *CDR2* and *MDR1*. #1 and #17 are from a series of 17 oral isolates from a single HIV positive patient [45]. *C. albicans* DSY1050 (from D. Sanglard [19]) is a FLC hyper-susceptible strain containing homozygous deletions of *CDR1*, *CDR2* and *MDR1*. *Cryptococcus neoformans* H99 (from J. Lodge; [46]), *Candida krusei* (our collection; [47]) and *Saccharomyces cerevisiae* W303 (our collection; [48]) were all used to determine if other fungal species are capable of importing [^3^H]-FLC. A collection of 35 un-matched clinical isolates (from D. Stevens; [34] were used to determine import of [^3^H]-FLC in isolates with known and unknown mechanisms of resistance. The *Saccharomyces cerevisiae* haploid gene-deletion library was originally obtained from Research Genetics (Huntsville, AL).

Strains were maintained on YEPD (10 g of yeast extract, g of peptone, 20 g of dextrose, with or without 15 g of Bacto Agar per liter), or on CSM complete medium (0.75 g CSM [Bio 101; Vista, CA], 1.7 g yeast nitrogen base without amino acids or ammonium sulfate, 5 g ammonium sulfate, 20 g dextrose per liter). All isolates were stored at −80°C in CSM or YEPD containing 10% glycerol. Overnight cultures were inoculated from a single colony on a YEPD agar plate and inoculated into YEPD broth and grown overnight at 30°C, 180 rpm, unless otherwise noted.

### Materials and Drugs

Medium components were obtained from Fischer Scientific (Pittsburgh, PA) or Bio 101 (Vista, CA). General chemicals were obtained from Fisher Scientific, or Sigma-Aldrich (St. Louis, MO). Itraconazole, voriconazole, ketoconazole, paclobutrazol, azaconazole, flucytosine and R6G were obtained from Sigma-Aldrich (St. Louis, MO). FLC was a generous donation from Pfizer, New York, NY. Fluorescein-labeled ITC was the generous gift of J. Lui and collaborators (Johns Hopkins U). POS, benznidazole, Tipifarnib, Tipifarnib 2g, and STN54 were the generous gifts of Fred Buckner, (U Washington). Oligopeptides were obtained from Neo BioScience (www.neobiosci.com).

### Fluconazole Uptake by Fungal Cells

FLC uptake was determined using [^3^H]-FLC (specific activity 740 GBa/mmol, 20 Ci/mmol, 2×10^4^ CPM/pmol, 1 uCi/uL; 50 uM FLC; custom synthesis by Amersham Biosciences, UK). Cells were grown overnight in CSM complete medium at 30°C to a density typically between OD_600_ 6.0 to 8.0, unless otherwise noted. Cells were subsequently harvested by centrifugation (3000×g, 5 m) and washed three times with YNB complete (1.7 g yeast nitrogen base without amino acids or ammonium sulfate, 5 g ammonium sulfate per liter, pH 5.0) without glucose (for starvation) and without supplementation, unless otherwise noted. Cells were resuspended at an OD_600_ of 75 in YNB for 2–3 h for glucose starvation. Reaction mixes consisted of 250 uL of YNB, 200 uL of cells (75 OD) and 50 uL of [^3^H]-FLC (1/100 dilution of stock). The resulting [^3^H]-FLC concentration is 50 nM (0.015 ug/ml), which is significantly below the MIC for all strains. Samples (100 ul) were removed at various time points and placed into 5 ml stop solution (YNB +20 µM [6 ug/ml] FLC), filtered on glass fibre filters (24 mm GF/C; Whatman; Kent, UK) pre-wetted with stop solution and washed with 5 ml of stop solution. Filters were transferred to 20 ml scintillation vials. Scintillation cocktail (Ecoscint XR, National Diagnostics, Atlanta GA) was added (15 ml) and the radioactivity associated with the filter was measured with a liquid scintillation analyzer (Tri-Carb 2800 TR; Perkin Elmer; Waltham, MA) and normalized to CPM/1×10^8^ cells. Rate of [^3^H]-FLC uptake was determined by incubating samples in the presence of increasing concentrations of unlabeled FLC (unless otherwise noted) and samples were analyzed for [^3^H]-FLC accumulation at designated time points. These data were analyzed using linear regression to determine the rate of [^3^H]-FLC uptake. GraphPad Prism 4.0 was used to determine linear regression, Michaelis-Menten import kinetics (*V_max_* and *K_m_*) and 50% inhibitory concentration (IC_50_) values.

Uptake of [^3^H]-FLC by a collection of 35 *Candida* clinical isolates, and by the *Saccharomyces* gene deletion library was determined by following the above protocol with the exception that cells (1.5 ml) were grown for 48 h in 2.0 ml 96-deep well plates (Masterblock; Greiner bio-one; Monroe, NC). The reactions were half of the size: 125 uL of YNB, 100 uL of cells, and 25 uL of [^3^H]-FLC (1/100 dilution of stock). Samples (100 ul) from the reaction were removed 24 h post incubation and filtered over 96-well multiscreen HTS filter plates (Opaque non-sterile with lid, 1.2 µm glass fibre type C filter; Millipore, Billerica, MA), wells were dried and the bottoms were sealed with sealing tape (Perkin Elmer; Waltham, MA). Scintillation fluid (150 ul) was added to each well, the tops were sealed with sealing tape and the plates were counted on a 96-well liquid scintillation counter using both top and bottom counting (Liquid Scintillation and Luminescence Counter, WALLAC/Jet, 1450 Microbeta).

### FACS Analysis of R6G Efflux

Isolates were grown to exponential phase in 5 ml YEPD at 30°C with 180 RPM shaking. Cells were collected by centrifugation (3000×g 5 m) and washed three times in sterile water. Cells were resuspended to OD600 = 0.4 in 50 mM phosphate buffer pH 6.0 with 5 mM 2-deoxy-D-glucose. Cells were incubated for 60 m at 30°C with shaking. R6G was added to a final concentration of 10 µm and cells were incubated for 90 minutes at 30° with shaking. Cells were collected by centrifugation and washed twice in 50 mM phosphate buffer pH 6.0. Cells were resuspended to OD600 = 0.2 in 50 mM phosphate buffer pH 6.0. 500 µl of cells was diluted 1∶2 in 50 mM phosphate buffer pH 6.0, and accumulation was measured by fluorescence activated cell sorting (FACS). Glucose was added to a final concentration of 40 µm and efflux was measured by analyzing an aliquot of cells diluted 1∶10 in ice-cold 50 mM phosphate buffer pH 6.0 by FACS at the time intervals indicated using a Beckman Coulter EpicsXL-MCL 4-colour cell analyzer. The geometric mean of the fluorescence of each sample was calculated using FlowJo software.

### Characterization of FLC Import

Further studies were done to determine the effect of changes in the incubation conditions have on FLC import:

#### Heat and methanol killed cells

Uptake of [^3^H]-FLC was determined in cells inactivated (killed) by heat (70°C for 45 m) and methanol (95% methanol for 45 m). These conditions decrease colony forming units greater than 100 fold compared to the untreated culture. To assess permeability, cells were stained using the Live/Dead FungaLight Yeast Viability Kit (Invitrogen), which contains SYTO9 and propidium iodide. The stained cells were analyzed by FACS (fluorescent activated cell sorting). After killing, cells were harvested by centrifugation, washed with YNB complete and resuspended at original density of OD_600_ 75. Samples were compared to live SC5314 cells.

#### Temperature

Cells grown overnight as previously discussed, then incubated at 4°C, 25°C, 30°C or 37°C for various times and samples were removed and processed as described.

*pH*: Cells were incubated in YNB without supplementation adjusted to pH 3, 5, 7 or 9 with potassium hydroxide (to increase pH) or hydrochloric acid (to decrease pH) and processed at various timepoints as outlined previously. Samples were compared to pH 5 and recorded as relative change.

#### Growth phase

For each growth phase tested (log, late log, post diauxic shift) cells were inoculated into YEPD broth at varying concentrations and grown overnight. After overnight growth, log phase cultures were harvested at OD_600_ 0.4, late log at OD_600_ 2.0 and post diauxic shift at OD_600_ 8–10. Samples were harvested by centrifugation (3000×g, 5 m) and processed as previously described. All samples were compared to the post diauxic shift sample and recorded as relative change. Post diauxic shift samples were used for most of the analyses as it reduces the sample volumes that must be processed for the analyses.

#### Hyphal cells

SC5314 cells were grown overnight in YEPD (30°C, 180 rpm). Cells were then washed three times with sterile, distilled H_2_0. Cells were diluted to a concentration three logs below their overnight YEPD density in spider medium (10 g nutrient broth, 10 g mannitol, 2 g dibasic K_2_HPO_4_ per liter) pre-warmed to 37°C. Cells were then incubated at 37°C without shaking until germ tubes formed (approximately 1 h). Hyphae were washed two times with sterile, distilled H_2_0 and resuspended in YNB complete (no glucose) for 2 h until they were processed and analyzed for uptake. Samples were compared to SC5314 yeast cells.

#### Low oxygen

SC5314 cells were grown over night in YEPD. In order to create a microaerophilic environment, a layer of mineral oil was added to the surface of the sample and cultures grown at 30°C without shaking, as previously described [49]. Samples were compared to SC5314 cultures at 30°C.

## Supporting Information

Figure S1 Structures of compounds from Table 3. The structures were drawn using freeware MarvinSketch v5.3.3. Most of the structures were based on the compound structures in the NIH PubChem Compound Database [49]. The structure of the fluorescein labeled ITC in Table 3 is very similar to ITC, but the exact structure has not been confirmed.
(0.22 MB TIF)
