# Supplementary figures and images for: Azole Drugs Are Imported By Facilitated Diffusion in Candida albicans and Other Pathogenic Fungi
Source: PLoS Pathog. 2010 Sep 30;6(9):e1001126. doi: 10.1371/journal.ppat.1001126 (PMC2947996; doi:10.1371/journal.ppat.1001126)

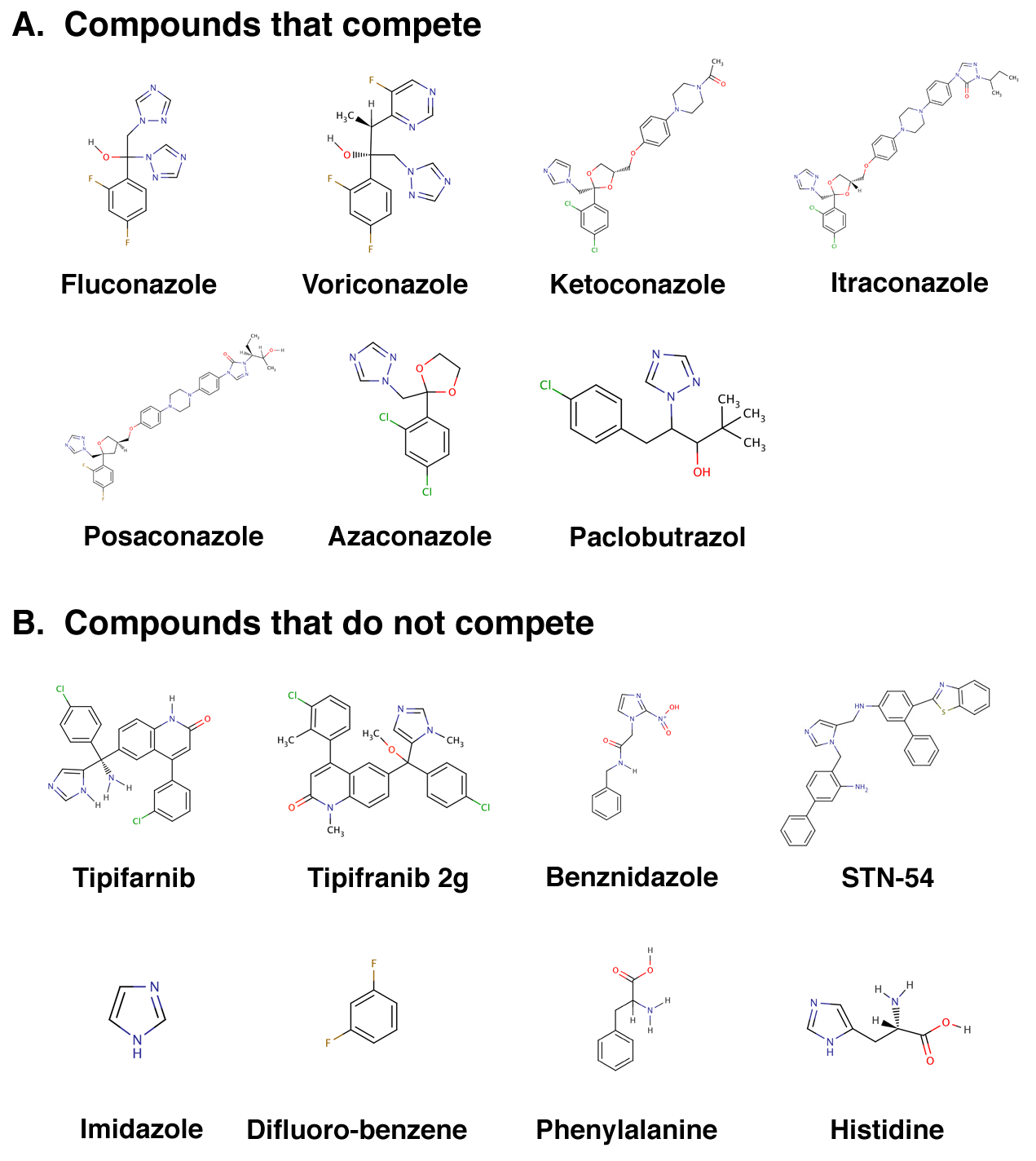

Supplement: Figure S1 — Structures of compounds from Table 3. The structures were drawn using freeware MarvinSketch v5.3.3. Most of the structures were based on the compound structures in the NIH PubChem Compound Database [49]. The structure of the fluorescein labeled ITC in Table 3 is very similar to ITC, but the exact structure has not been confirmed. (0.22 MB TIF) [file ppat.1001126.s001.tif]
